# Survival Benefit of Metformin as an Adjuvant Treatment for Head and Neck Cancer: A Systematic Review and Meta-Analysis

**DOI:** 10.3389/fphar.2022.850750

**Published:** 2022-05-13

**Authors:** Yu Jiao, Dongjuan Liu, Yi Sun, Zitong Chen, Sai Liu

**Affiliations:** ^1^ The First Affiliated Hospital of China Medical University, Shenyang, China; ^2^ Liaoning Provincial Key Laboratory of Oral Diseases, School and Hospital of Stomatology, China Medical University, Shenyang, China

**Keywords:** metformin, head and neck cancer, meta-analysis, therapy, survival

## Abstract

**Background/Aims:** The relationship between the efficacy of metformin and the prognosis of patients with head and neck cancer (HNC) was still unclear. This study aims to clarify the prognostic value of metformin treatment using meta-analysis.

**Methods:** Studies related to HNC prognosis and metformin were searched in Cochrane Library, Embase, LILACS, MEDLINE and PubMed databases. A meta-analysis was performed to evaluate the association between metformin therapy and the prognosis of HNC on overall survival (OS), disease-free survival (DFS) and disease-specific survival (DSS) and whether article quality, comorbidities, age, region or smoking had an influence on the prognosis of metformin treatment. Pooled hazard ratio (HR) and 95% confidence interval (CI) were analyzed to assess the effect.

**Results:** Eleven eligible studies involving 14,694 participants were included. Metformin increased the OS (HR = 0.87, 95% CI: 0.76–0.99), but failed on DFS (HR = 0.67, 95% CI: 0.40–1.09) or DSS (HR = 0.69, 95% CI: 0.41–1.14) in HNC patients. Subgroup analysis showed metformin was associated with improved OS (HR = 0.66, 95% CI: 0.49–0.88), DFS (HR = 0.49, 95% CI: 0.26–0.92) and DSS (HR = 0.38, 95% CI: 0.22–0.65) in studies with higher Newcastle-Ottawa Scale (NOS) scores. Subgroup analysis of age indicated that patients younger than 65 years (OS, HR = 0.67, 95% CI: 0.49–0.92) were more likely to benefit from metformin treatment. Subgroup analysis of comorbidities showed metformin significantly improved patient outcomes in studies without adjusted for comorbidities (OS, HR = 0.66, 95% CI: 0.51–0.85; DSS, HR = 0.38, 95% CI: 0.22–0.65), but not in studies that adjusted for comorbidities.

**Conclusions:** Metformin improved the prognosis of HNC patients as an adjuvant therapy, especially in those with higher NOS scores. Age and comorbidities of HNC patients influenced the therapeutic effect of metformin. Further well-conducted investigations are needed.

## Introduction

Head and neck cancer (HNC) is used to describe cancers that occur in the larynx, nasal cavity, oral cavity, paranasal sinuses, throat, as well as salivary glands. Most HNCs are squamous cell carcinomas (Lutzky et al., 2008). HNC is one of the most common types of cancer, with approximately 900,000 new cases and 450,000 related deaths worldwide per year (Sung et al., 2021). The prognosis of patients with HNC remains unsatisfactory, despite advances in surgery, chemotherapy and radiotherapy. Recently, several hypoglycemic drugs have been found to be able to reduce the risk of cancer and have a positive effect on cancer treatment (Shlomai et al., 2016). Among hypoglycemic agents, metformin has attracted much attention as the most potential anti-cancer therapeutic assistant (Morales and Morris 2015; Vilaseca et al., 2020).

Metformin has shown a preventive effect on a variety of cancer types, including pancreatic cancer, colorectal cancer and hepatocellular carcinoma, reducing the incidence and mortality (Wan et al., 2018; Schulte et al., 2019; Ng et al., 2020). Metformin has been repeatedly reported to decrease the risk of HNC (Becker et al., 2014; Tseng 2014; Yen et al., 2015; Tseng 2016; Lerner et al., 2017; Tseng 2018; Veitz-Keenan et al., 2019; Mekala et al., 2020), but its role in prolonging NHC patient survival remains controversial. In 2020, there was a meta-analysis including seven retrospective cohort studies and exploring the correlations between metformin and HNC patient survival (Wang et al., 2020). The results showed that there was no significant association between the use of metformin and survival of HNC patients (HR = 0.89, 95% CI: 0.66–1.18; *p* = 0.413). However, this meta-analysis missed three studies (Spratt et al., 2016; Chang et al., 2017; Ogunsakin et al., 2018), which may lead to a biased analysis result. In addition, after this meta-analysis, a new study on the correlations between metformin and the survival of HNC patients was published (Hu et al., 2020). Consequently, further meta-analysis is required. To the end, we performed a meta-analysis on 11 cohort studies in order to determine the effect of metformin on HNC patient survival. Furthermore, subgroup analysis was also performed.

## Methods

### Systematic Literature Search

Research studies were obtained from Cochrane Library, Embase, LILACS, MEDLINE and PubMed databases, without time or language restrictions.

The search strategy for PubMed included the following terms: head and neck squamous cancer [title] OR head and neck squamous carcinoma [title]OR head and neck cancer [title] OR head and neck carcinoma [title] OR HNSCC [title] OR nasopharyngeal [title] OR oral squamous cell carcinoma [title] OR laryngeal [title] AND metformin [title] AND adjuvant [Any field]. Cited studies of the included studies was also checked. The search was performed on 3 May2021.

### Eligibility Criteria

The following criteria were used to select randomized controlled trials and observational studies: participants, outcomes, and study design (PICOS). The inclusion criteria were defined as follows: 1) The population is patients with head and neck cancer; 2) Metformin is used as adjuvant therapy; 3) Metformin is associated with head and neck cancer; 4) Clinical research. The exclusion criteria were defined as follows: 1) Cell culture and animal experiments; 2) Article does not contain survival data; 3) Published not in English; 4) Data overlap.

The literature retrieval and screening were conducted independently by two authors (Jiao and Sun). If there is an unidentified document between the two authors, the third author (Chen) will make a decision based on the inclusion criteria.

### Data Collection Process and Data Items

An author (YJ) selected the required information from the selected studies: author, publication year, country, sample size, study design, stage/location, the mean or median age of metformin, outcomes, definition of metformin exposure, follow-up (months), adjusted variables, results and conclusions. If there was no original data required for meta-analysis in the article, we sent an email to the corresponding author or calculated from the data extracted from Kaplan-Meier curves using the method given by Jayne F Tierney (Tierney et al., 2007). The second author (DJL) cross-checked all the retrieved information. Likewise, any disagreements were resolved through discussion and mutual agreement between the two authors. The third author (ZDC) participated in making the final decision when needed.

It is worth noting that: first, in Yung-An Tsou’s article, the number of people in the metformin treatment group and the control group in the abstract was inconsistent with the number in the results. After communicating with the author, the correct value was confirmed (Tsou et al., 2019). Second, Xin Hu’s article did not give HR values (Hu et al., 2020), and the corresponding HR and CI values were calculated from the data extracted from Kaplan-Meier curves using the method given by Jayne F Tierney (Tierney et al., 2007). Third, although the HR values were not directly provided in Amie Ogunsakin’s article (Ogunsakin et al., 2018) and Pei Hun Chang’s article (Chang et al., 2017), accurate HR and CI values can be calculated from the data in the articles.

### Quality Assessment

Newcastle-Ottawa Scale (NOS) was used to evaluate the quality of non-randomized studies (Stang 2010; Lo et al., 2014). The quality of studies was independently assessed by two authors (YJ and DJL). Any differences were discussed and resolved by the two authors or judged by the third author (YS).

### Statistical Analysis

The summarized data was analyzed by Review manager 5.4 and Stata 12. HRs with 95% CIs were used to calculate the summary survival effect. The pooled analysis was performed by obtaining the corresponding HR and CI values. Heterogeneity was evaluated by the I^2^ statistics. I^2^ values of 25%, 50%, and 75% were considered as low, moderate, and high heterogeneity, respectively (Higgins and Thompson 2002). By removing one study at a time, a sensitivity analysis was performed to assess the impact of each study on heterogeneity. Publication bias was intuitively assessed by examining the asymmetry of the funnel plot. In addition, Begg’s test was also used to evaluate publication bias (Hayashino et al., 2005; Peters et al., 2006). The probability value was bilateral, and *p* < 0.05 was considered statistically significant. Use Review manager 5.4 for data analysis.

## Results

### Literature Search

**FIGURE 1:**
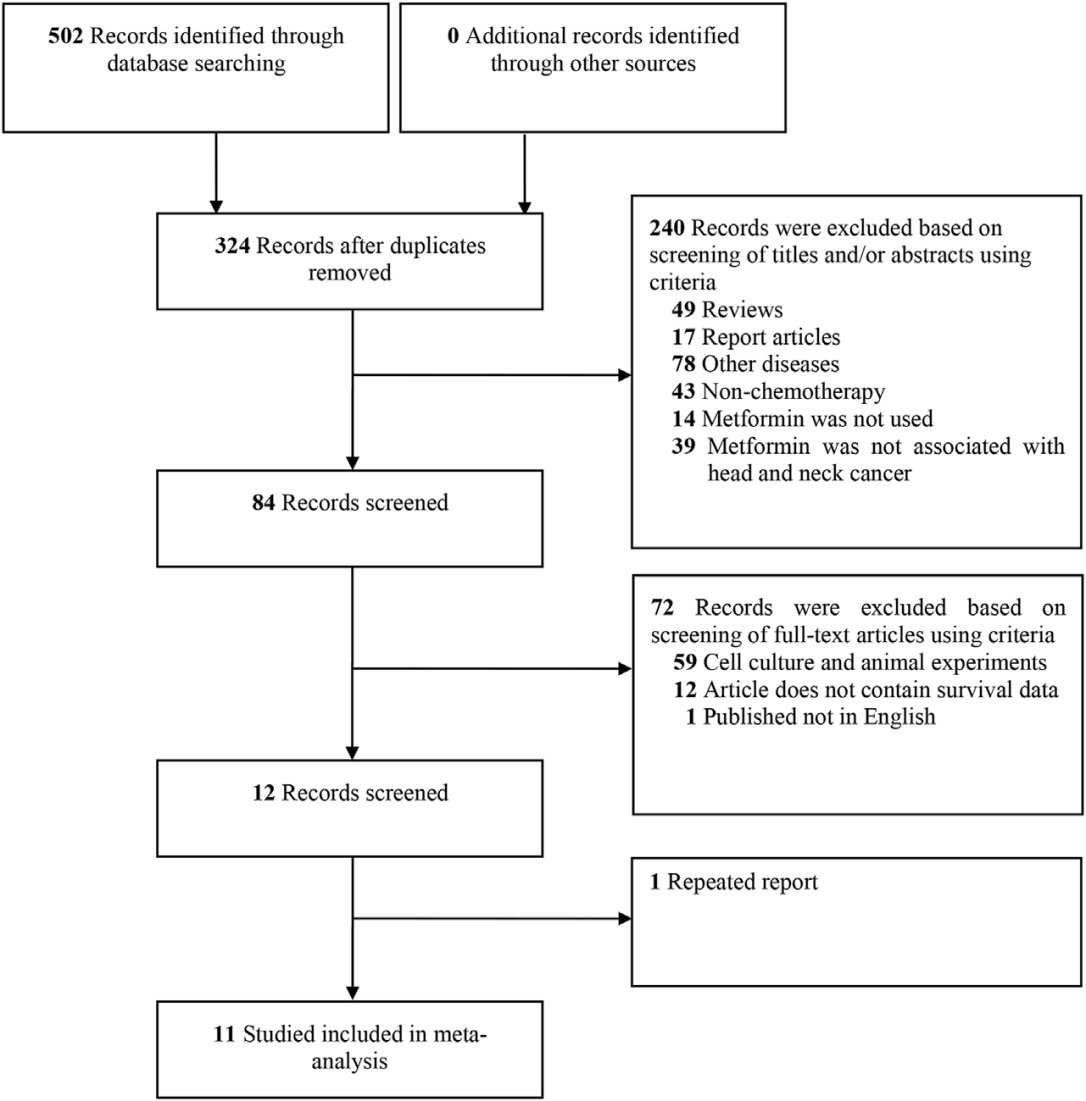
Flow diagram of studies search and selection criteria for systemic review and meta-analysis.

The selection process is shown in Figure 1. A total of 502 studies were identified from the Cochrane Library, Embase, LILACS, MEDLINE and PubMed databases initially. After screening the titles and abstracts, 84 studies were evaluated further for eligibility. Of the 84 studies, 72 did not meet the inclusion criteria: 59 studies belonged to cell culture or animal experiments; 12 studies could not contain survival data; one study was not English study; one study was data overlap. Thus, 11 cohort studies were included in this systematic review (Sandulache et al., 2014; Kwon et al., 2015; Spratt et al., 2016; Chang et al., 2017; Ogunsakin et al., 2018; Quimby et al., 2018; Stokes et al., 2018; Alcusky et al., 2019; Lee et al., 2019; Tsou et al., 2019; Hu et al., 2020).

### Study Characteristics

The summary characteristics of the included studies were shown in Table 1. Sample sizes ranged from 34 to 7,872 patients. All included studies were from 2014 to 2020, and all studies were published in English. Belonging to cohort studies, these studies were conducted in different countries: Canada (Quimby et al., 2018; Lee et al., 2019), China (Chang et al., 2017; Tsou et al., 2019; Hu et al., 2020), the United States (Sandulache et al., 2014; Spratt et al., 2016; Ogunsakin et al., 2018; Stokes et al., 2018), Europe (Alcusky et al., 2019) and South Korea (Kwon et al., 2015). All selected studies are clinical studies and belong to cohort studies. In these 11 studies, exposure to metformin was given different definitions.

**TABLE 1:** A summary of characteristics of the included studies.

First author, Year	Study characteristics					Definition of metformin exposure	Follow-up (months)	Adjusting variables	Results
	Country	Sample size (met/total)	Design	Stage/Location	Mean or median age (years)				
Hu et al. (2020)	China	44/88	Cohort	OSCC	Mean 53.50 ± 9.53	Met alone or in combination with other drugs	60	Age, gender, urban and rural residence, smoking, alcohol, betel quid chewing, location, TNM stage	RFS:0.42 (0.27–0.64)
Lee et al. (2019)	Canada	195/329	Cohort	OC, OP, LX	Mean 67.3 ± 9.8	Taking met at the time of presentation	37.2	Primary site, age, treatment modality, extra-capsular spread, perineural invasion, CCI score, smoking, alcohol, Follow-up time, TNM stage	OS:1.04 (0.72–1.5); RFS:1.04 (0.66–1.62); DSS:1.16 (0.68–1.98)
Tsou et al. (2019)	China	49/141	Cohort	HP	Mean 66.45	Previous OHA and persisted though the CCRT treatment until the latest follow up	48	Age, alcohol, betel nut, cigarette, TNM stage or disease stage	OS: Early stage: 1.54 (0.32–7.22), Late stage: 0.23 (0.08–0.68); DFS: Early stage: 1.44 (0.09–2.14); Late stage: 0.23 (0.07–0.68)
Alcusky et al. (2019)	Italy	708/7,872	Cohort	NM	Median 68.1 (59.3, 76.7)	Exposed to met after HNC diagnosis	35.2	Gender, age, location of residence, mean-centred calendar time in year, TD exposure to chemotherapy indicator, TD exposure to radiation therapy indicator, TD tumor resection indicator, TD diagnosis of regional and metastatic disease indicator, and ECS	OS:0.81 (0.61–1.09)
Quimby et al. (2018)	Canada	165/1,231	Cohort	NP, HP, LX	Mean 74.55 ± 6.09	Taking met at the time of diagnosis	36	Age, gender, ECS, treatment type, primary site	OS: 1.10 (0.86–1.41); DSS:1.00 (0.70–1.44)
Stokes et al. (2018)	United States	124/1,646	Cohort	OC, OP and other	>66, Mean 73.99^*^	Start within 6 months after diagnosis	24	Gender, age, race, marital status, SEER registry, population density, TNM stage	OS:0.74 (0.50 1.09); CSS: 0.33 (0.16 0.67)
Ogunsakin et al. (2018)	United States	11/34	cohort	LX, OP	Not given	Taking met for at least 1 year and at least 1 year after the pathological diagnosis of HNC or death within 1 year after diagnosis, and must take met before death	60	Age, primary cancer treatment, race, glucose control, age at death and tumor stage of SCC	OS:0.42 (0.1–1.74)
Chang et al. (2017)	China	39/252	Cohort	III, IVA, IVB	Mean 56.1 ± 12.2	Received met at the time of definitive diagnosis of cancer	24	Age, gender, disease stage, TNM stage, Eastern Cooperative Oncology Group performance status, CCI, BMI, smoking, alcohol, betel quid chewing	OS:0.79 (0.44–1.42); RFS:1.02 (0.6–1.74)
Spratt et al. (2016)	United States	102/1745	Cohort	OP	Median 61 (35–79)	From time of diagnosis continued for a minimum of 5 years (or death or last follow-up if less than 5 years)	60	Age, gender, primary site of disease, TNM stage, smoking, HPV, P16, dose, chemotherapy	OS:0.73 (0.4–1.33)
Kwon et al. (2015)	Korea	99/1,151	Cohort	NM	Median 61 (20–80)	6 months before HNSCC diagnosis through 1 month after diagnosis, and at least 1 month after diagnosis	65.1	Patient age and gender, site, TNM stage, smoking status, alcohol, BMI, and initial treatment modalities	OS:0.7 (0.4–1.22); CSS:0.45 (0.20–0.99)
Sandulache et al. (2014)	United States	21/205	Cohort	LSCC	Mean 64	Taking metformin during treatment	>36	Age, gender, race, smoking, alcohol consumption, TNM stage	OS:0.34 (0.12–0.96); DFS:0.50 (0.21–1.22)

*The mean year was calculated from data.

Abbreviations: met = metformin; OS, overall survival; DFS, disease-free survival; DSS, disease-specific survival; RFS, recurrence-free survival; CSS, cancer-specific survival; OSCC, oral squamous cell carcinoma; OC, oral cavity; OP, oropharynx; LX, larynx; HP, hypopharynx; NP, nasopharynx; NM, not mentioned; LSCC, laryngeal squamous cell carcinoma; HNSCC, head and neck squamous cell carcinoma; TD, time-dependent; CCI, charlson comorbidity index; ECS, elixhauser comorbidity score; NOS, Newcastle-Ottawa Scale.

Four studies defined metformin exposure as the use of metformin when diagnosing head and neck cancer (Chang et al., 2017; Quimby et al., 2018; Alcusky et al., 2019; Lee et al., 2019), four studies defined metformin exposure as continuous use of metformin during treatment (Sandulache et al., 2014; Spratt et al., 2016; Tsou et al., 2019; Hu et al., 2020), one study defined metformin exposure as the use of metformin within 6 months after diagnosis (Stokes et al., 2018), and one study defined metformin exposure as having to take metformin for at least one year and at least one year after the pathological diagnosis of HNC or death within one year after diagnosis, and must take metformin before death (Ogunsakin et al., 2018). In addition, another study divided the definition of metformin exposure into three categories: prediagnosis (>6 months prediagnosis), per-diagnosis (6 months before HNC diagnosis through 1 month after diagnosis), and postdiagnosis (>1 month after diagnosis) (Kwon et al., 2015). In the analysis of the research outcomes, three indicators: overall survival (OS) (Sandulache et al., 2014; Kwon et al., 2015; Spratt et al., 2016; Chang et al., 2017; Ogunsakin et al., 2018; Quimby et al., 2018; Stokes et al., 2018; Alcusky et al., 2019; Lee et al., 2019; Tsou et al., 2019), disease-free survival (DFS) (Sandulache et al., 2014; Chang et al., 2017; Lee et al., 2019; Tsou et al., 2019; Hu et al., 2020), and disease-specific survival (DSS) (Kwon et al., 2015; Quimby et al., 2018; Stokes et al., 2018; Lee et al., 2019) were selected. The impact of confounding factors on the results was considered.

### Quality of Studies

The risk of bias of the studies was accessed by NOS (Lo et al., 2014). The NOS scores of included studies were shown in Table 2. Of the 11 studies, the NOS scores of two studies were six, five studies were seven, and the remaining four studies were eight. The average score calculated for NOS is 7.18.

**TABLE 2:** NOS scores of included studies.

First author, Year	Selection				Comparability	Outcomes			Scores
	Representativeness of exposed cohort	Selection of non-exposed cohort	Ascertainment of exposure	Demonstration that outcomes of interest was not present at start of study	Comparability of cohorts on the basis of the design or analysis	Assessment of outcomes	Was follow-up long enough for outcomes to occur	Adequacy of follow-up of cohorts	
Hu Xin et al. (2020)	☆	☆	—	☆	☆☆	—	☆	☆	7
Lee et al. (2019)	☆	☆	—	☆	☆	☆	☆	☆	7
Tsou et al. (2019)	☆	☆	☆	☆	☆	☆	☆	☆	8
Alcusky et al. (2019)	☆	☆	☆	☆	☆	—	☆	☆	7
Quimby et al. (2018)	☆	☆	☆	☆	☆	☆	—	—	6
Stokes et al. (2018)	☆	☆	☆	☆	☆☆	☆	—	☆	8
Ogunsakin et al. (2018)	☆	☆	☆	☆	☆	—	☆	☆	7
Chang et al. (2017)	☆	☆	—	☆	☆☆	☆	—	☆	7
Spratt et al. (2016)	☆	☆	—	☆	☆	—	☆	☆	6
Kwon et al. (2015)	☆	☆	—	☆	☆☆	☆	☆	☆	8
Sandulache et al. (2014)	☆	☆	—	☆	☆	☆	☆	☆	8

### Results of Individual Studies

**FIGURE 2:**
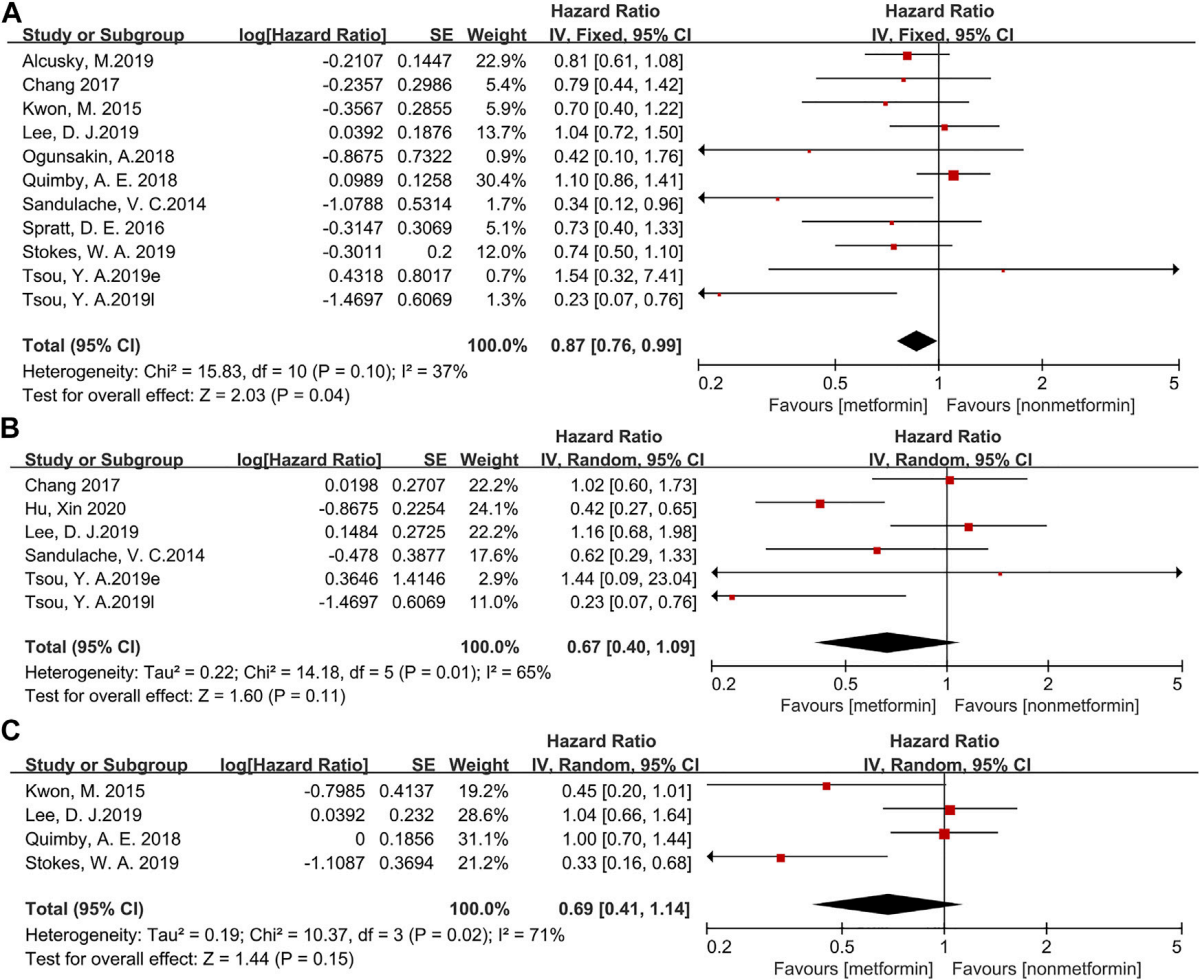
The forest plot of head and neck cancer according to metformin use for overall survival **(A)**, disease-free survival **(B)**, disease-specific survival **(C)**.

The forest map of survival data is shown in Figure 2. To evaluate the outcome, OS (13,311 patients) was used in ten studies; DFS (972 patients) was used in five studies; and DSS (4,355 patients) was used in four studies. When applying the fixed effects model, OS (HR = 0.87, 95% CI: 0.76–0.99, *p* = 0.04, Figure 2A) shows that metformin is beneficial to the treatment of head and neck cancer, but DFS (HR = 0.67, 95% CI: 0.40–1.09, *p* = 0.11, Figure 2B) and DSS (HR = 0.69, 95% CI: 0.41–1.14, *p* = 0.15, Figure 2C) on the random effects model do not indicate a significant effect of metformin in HNC patients.

### Sensitivity Analysis

The heterogeneity of OS (*χ* ^2^ = 15.83, df = 10, *p* = 0.10, *I* ^2^ = 37%) was obvious. A sensitivity analysis was performed by deleting one study at a time to evaluate the impact of each study on heterogeneity. Alexandra E Quimby’s article was considered to be the main reason for the high heterogeneity. After excluding this article, the heterogeneity was reduced (*χ* ^2^ = 10.61, df = 9, *p* = 0.30, *I* ^2^ = 15%), and the results were not greatly affected (HR = 0.78, 95% CI: 0.66–0.92, *p* = 0.003).

### Subgroup Analysis

The results of the subgroup analysis are demonstrated in Table 3.

**TABLE 3:** Results of subgroup analysis.

Sub-group	Number of effects	Heterogeneity	Subgroup analysis		
		*I* ^2^ (%)	HR	95% CI	*p* value
OS					
Age					
≥65 years old	6	51	0.88	0.69–1.13	0.33
<65 years old	5	0	0.67	0.49–0.92	0.01
Region					
Asian	4	33	0.69	0.47–1.00	0.05
North America and Europe	7	38	0.90	0.78–1.04	0.16
Quality of studies					
NOS>7.18	5	34	0.66	0.49–0.88	0.005
NOS<7.18	6	4	0.94	0.81–1.09	0.42
Adjusted for comorbidity					
Yes	4	7	0.97	0.82–1.13	0.66
No	7	8	0.66	0.51–0.85	0.002
Adjusted smoking					
Yes	6	49	0.79	0.61–1.02	0.07
No	5	26	0.90	0.77–1.06	0.2
DFS					
Age					
≥65 years old	3	67	0.67	0.19–2.32	0.52
<65 years old	3	68	0.64	0.36–1.13	0.13
Region					
Asian	4	67	0.55	0.27–1.12	0.1
North America and Europe	2	43	0.90	0.49–1.65	0.73
Quality of studies					
NOS>7.18	2	20	0.49	0.26–0.92	0.03
NOS<7.18	2	81	0.73	0.55–0.97	0.03
Adjusted for comorbidity					
Yes	3	81	0.78	0.40–1.51	0.46
No	3	20	0.48	0.22–1.03	0.06
Adjusted smoking					
Yes	5	64	0.59	0.32–1.06	0.08
No	1	—	1.02	0.60–1.73	0.94
DSS					
Age					
≥65 years old	3	75	0.76	0.43–1.34	0.34
<65 years old	1	—	0.45	0.20–1.01	0.05
Region					
Asia	1	—	0.45	0.20–1.01	0.05
North America and Europe	3	75	0.76	0.43–1.34	0.34
Quality of studies					
NOS>7.18	2	0	0.38	0.22–0.65	0.0004
NOS<7.18	2	0	1.02	0.76–1.35	0.92
Adjusted for comorbidity					
Yes	2	0	1.02	0.76–1.35	0.92
No	2	0	0.38	0.22–0.65	0.0004
Adjusted smoking					
Yes	2	68	0.73	0.33–1.65	0.45
No	2	86	0.6	0.20–1.78	0.36

Abbreviations: OS, overall survival; DFS, disease-free survival; DSS, disease-specific survival; NOS, Newcastle-Ottawa Scale.

The average score of NOS was 7.18. Studies with NOS > 7.18 were considered to belong to the high-quality group, and studies with NOS < 7.18 were classified as low-quality group. Four of the 11 studies were classified into the high-quality group (Sandulache et al., 2014; Kwon et al., 2015; Stokes et al., 2018; Tsou et al., 2019). In the subgroup of high-quality, significantly better survival was observed in metformin group compared to the non-metformin group on OS (HR = 0.66, 95% CI: 0.49–0.88, *p* = 0.005), DFS (HR = 0.49, 95% CI: 0.26–0.92, *p* = 0.03) and DSS (HR = 0.38, 95% CI: 0.22–0.65, *p* = 0.0004).

Subgroup analysis based on comorbidity showed that metformin significantly improved patient outcomes in studies without adjusted for comorbidities (OS, HR = 0.66, 95% CI: 0.51–0.85, *p* = 0.002; DSS, HR = 0.38, 95% CI: 0.22–0.65, *p* = 0.0004), but not in studies that adjusted for comorbidities.

When analyzing populations in different regions, OS (HR = 0.69, 95% CI: 0.47–1.00, *p* = 0.05), DFS (HR = 0.55, 95% CI: 0.27–1.12, *p* = 0.1), and DSS (HR = 0.45, 95% CI: 0.20–1.01, *p* = 0.05) showed that although it was not statistically significant, metformin was associated with better prognosis only in Asian HNC patients.

Subgroup analysis with the 65-year-old showed a better prognosis in the metformin group in the population under 65-years-old (OS, HR = 0.67, 95% CI: 0.49–0.92, *p* = 0.01).

In addition, subgroup analysis of adjusted smoking indicated that although there was no statistically significant difference between the use and non-use of metformin on OS (HR = 0.79, 95% CI: 0.61–1.02, *p* = 0.07), DFS (HR = 0.59, 95% CI: 0.32–1.06, *p* = 0.08), DSS (HR = 0.73, 95% CI: 0.33–1.65, *p* = 0.45).

### Publication Bias

**FIGURE 3:**
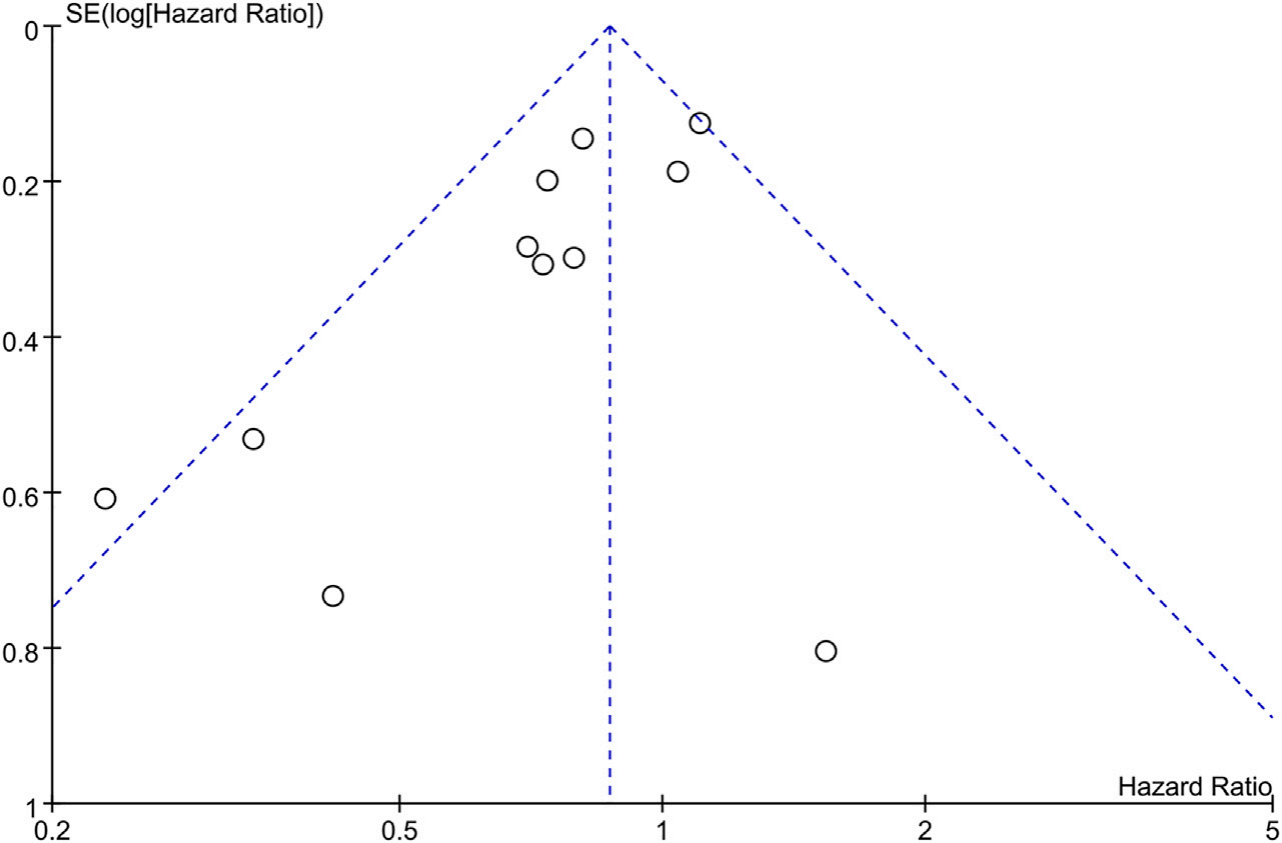
The funnel plot for overall survival.

The funnel plots (Figure 3) and Begg’s test (*p* = 0.119) suggested that there was no publication bias.

## Discussion

This systematic review and meta-analysis investigated the effect of metformin on HNC patients concerning survival. Our study demonstrated that metformin significantly prolonged the survival of HNC patients for the study of OS analysis. Especially, the survival benefit showed more obscure in studies with high-quality and among patients without comorbidity. In addition, it was worth noting that the efficacy of metformin therapy seems to be better for Asian HNC patients, although there was no statistical significance, there was a corresponding trend (*p* = 0.05).

The effect of metformin on prevention and treatment of HNC has been strengthened both by epidemiological data and laboratory studies. However, whether metformin is beneficial to the survival of HNC patients remains controversial. Wang *et al.* reported a meta-analysis of seven cohort studies of metformin treatment and HNC patients with unfavorable prognoses (Wang et al., 2020). Our results indicated that metformin significantly benefited the survival of HNC patients, which was different from the previous analysis. The difference in results may be related to the included participants. Our study included 11 studies with 14,694 participants, which is about five times the previous sample size. And the insignificance of DFS and DSS analysis of metformin on the survival of patients may also be connected with the small sample size.

Subgroup analysis based on quality showed that the results of OS, DFS and DSS all indicated that metformin significantly prolonged the survival of HNC patients in high-quality group. This result suggested that higher quality studies were more inclined to conclude that metformin was beneficial to patient survival. However, further high-quality studies with large sample sizes are needed.

Age-based subgroup analysis suggested that metformin significantly prolonged survival in HNC patients in people younger than 65 years. The results suggested that age might influence the effect of metformin, but more samples are needed to investigate.

Many HNC patients used metformin because of type 2 diabetes mellitus. In a subgroup analysis of comorbidities, mainly diabetes, metformin significantly improved patient outcomes in studies without adjusted for comorbidities (OS, HR = 0.66, 95% CI: 0.51–0.85, *p* = 0.002; DSS, HR = 0.38, 95% CI: 0.22–0.65, *p* = 0.0004), but not in studies that adjusted for comorbidities. This suggested that metformin use was associated with diabetes, and that HNC patients with previously diagnosed diabetes may have a better prognosis. However, the result made the impact of metformin on the prognosis of HNC patients confusing. Of the 11 studies, three were performed only in patients with type 2 diabetes (Ogunsakin et al., 2018; Tsou et al., 2019; Hu et al., 2020), and all three showed that metformin was associated with improved outcomes in HNC patients. However, there was no study performed only in patients without diabetes. In order to minimize the influence of diabetes on the prognosis of HNC patients with metformin, it is necessary to study the effect of metformin on the prognosis of patients without diabetes in the future.

Many reviews showed that metformin adjuvant treatment significantly associated with a survival benefit for Asian cancer patients (Tian et al., 2016; Wan et al., 2016; Wan et al., 2018). Subgroup analysis by region showed Subgroup analysis by region showed metformin was connected with longevity of Asian HNC patients despite no statistical significance. In addition, it was worth mentioning that there was an article that also showed that metformin can promote the prognosis of oral cancer patients (Huang et al., 2021). Because it was published in Chinese, this article was not included in this meta-analysis. Therefore, Asian HNC patients seemed more likely to benefit from metformin treatment. However, further epidemiological and laboratory studies are needed to understand these associations better.

Smoking is an important factor affecting the prognosis of patients with HNC. Subgroup analysis by adjusted smoking suggested that there was a trend that those who accepted metformin therapy lived longer than those who did not.

Several limitations of this study should be considered in this meta-analysis. First, only 11 cohort studies were included. Although we included more studies than that in the meta-analysis performed by Wang, it was still insufficient. Second, the influence of confounding factors on the results was beyond control. Third, measurement errors were unavoidable. Fourth, the dose and exposure time of metformin and the survival rate of HNC patients were unable to determine. The last, the effect of the interaction between metformin and other drugs or treatments on the outcome was unable to evaluate.

## Conclusion

This systematic review and meta-analysis showed that metformin had a significant improvement in overall survival of HNC patients, thus supporting metformin as an adjunct to the treatment of head and neck cancer. Especially, the efficacy of metformin in the high-quality group was more significant than that in the low-quality group. In a subgroup analysis of comorbidities, mainly diabetes, metformin significantly improved patient outcomes in studies without adjusted for comorbidities, but not in studies that adjusted for comorbidities. It is necessary to study the effect of metformin on the prognosis of HNC patients in the population without diabetes in the future. In addition, Asian HNC patients seemed to be more likely to benefit from the metformin therapy. However, the quantitative limitation of studies undermined the power of the analysis. There is a need for further studies with large sample sizes to investigate the relationship between metformin and the survival of HNC patients.

## Data Availability

The original contributions presented in the study are included in the article/Supplementary Material, further inquiries can be directed to the corresponding author.
